# (η^5^-Cyclo­penta­dien­yl)(propionitrile-κ*N*)bis­(triphenyl­phosphine-κ*P*)ruthenium(II) trifluoro­methane­sulfonate

**DOI:** 10.1107/S1600536809038720

**Published:** 2009-10-03

**Authors:** Klaus Mauthner, Karl Kirchner, Kurt Mereiter

**Affiliations:** a Institute of Applied Synthetic Chemistry, Vienna University of Technology, Getreidemarkt 9/163, A-1060 Vienna, Austria; b Institute of Chemical Technologies and Analytics, Vienna University of Technology, Getreidemarkt 9/164SC, A-1060 Vienna, Austria

## Abstract

The title compound, [Ru(C_5_H_5_)(C_3_H_5_N)(C_18_H_15_P)_2_]CF_3_SO_3_, forms yellow crystals with a distinctly hemimorphic habit. It contains a half-sandwich complex of ruthenium with a three-legged piano-stool geometry, with Ru—P = 2.3585 (4) and 2.3312 (4) Å, and Ru—N = 2.0422 (15) Å as the legs. The CF_3_SO_3_^−^ anion is anchored in the crystal lattice by C—H⋯O and C—H⋯F hydrogen bonds, with C⋯F,O distances starting at 3.125 (2) Å.

## Related literature

For the application of nitrile-substituted ruthenium–cyclo­penta­dienyl complexes in catalysis, see: Trost *et al.* (2001 ▶). For the synthesis, chemistry and crystal structures of related ruthenium–cyclo­penta­dienyl complexes, see: Carreón *et al.* (1997 ▶); Cordiner *et al.* (2003 ▶); Mauthner *et al.* (1999 ▶); Rüba *et al.* (1999 ▶, 2002 ▶); Bruce *et al.* (1982 ▶).
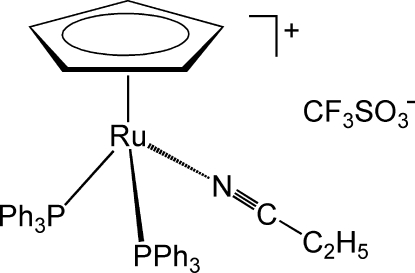


## Experimental

### 

#### Crystal data

[Ru(C_5_H_5_)(C_3_H_5_N)(C_18_H_15_P)_2_]CF_3_SO_3_
*M* *_r_* = 894.85
Monoclinic, 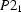

*a* = 9.9991 (4) Å
*b* = 17.2172 (7) Å
*c* = 11.4605 (5) Å
β = 92.126 (1)°
*V* = 1971.64 (14) Å^3^
*Z* = 2
Mo *K*α radiation
μ = 0.59 mm^−1^
*T* = 123 K
0.31 × 0.21 × 0.20 mm

#### Data collection

Bruker SMART APEX CCD diffractometer
Absorption correction: multi-scan (*SADABS*; Bruker, 2003 ▶) *T*_min_ = 0.79, *T*_max_ = 0.89
29537 measured reflections
11391 independent reflections
11056 reflections with *I* > 2σ(*I*)
*R*_int_ = 0.023

#### Refinement

*R*[*F* ^2^ > 2σ(*F* ^2^)] = 0.025
*wR*(*F* ^2^) = 0.061
*S* = 1.02
11391 reflections
505 parameters
1 restraint
H-atom parameters constrained
Δρ_max_ = 0.83 e Å^−3^
Δρ_min_ = −0.24 e Å^−3^
Absolute structure: Flack (1983 ▶), 5488 Friedel pairs
Flack parameter: −0.038 (12)

### 

Data collection: *SMART* (Bruker, 2003 ▶); cell refinement: *SAINT* (Bruker, 2003 ▶); data reduction: *SAINT*, *SADABS* and *XPREP* (Bruker, 2003 ▶); program(s) used to solve structure: *SHELXS97* (Sheldrick, 2008 ▶); program(s) used to refine structure: *SHELXL97* (Sheldrick, 2008 ▶); molecular graphics: *SHELXTL* (Sheldrick, 2008 ▶); software used to prepare material for publication: *SHELXTL*.

## Supplementary Material

Crystal structure: contains datablocks global, I. DOI: 10.1107/S1600536809038720/ez2188sup1.cif

Structure factors: contains datablocks I. DOI: 10.1107/S1600536809038720/ez2188Isup2.hkl

Additional supplementary materials:  crystallographic information; 3D view; checkCIF report

## Figures and Tables

**Table 1:** Hydrogen-bond geometry (Å, °)

*D*—H⋯*A*	*D*—H	H⋯*A*	*D*⋯*A*	*D*—H⋯*A*
C1—H1⋯F1	0.95	2.49	3.125 (2)	125
C3—H3⋯O1^i^	0.95	2.50	3.154 (3)	126
C7—H7*A*⋯O3	0.99	2.43	3.346 (3)	153
C28—H28⋯O1^i^	0.95	2.56	3.328 (2)	138
C44—H44⋯O2	0.95	2.58	3.209 (2)	124

Symmetry code: (i) 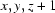.

**Fig. 1.**
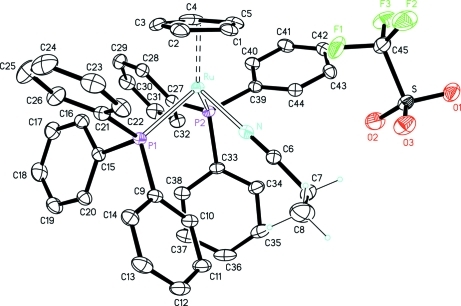
Perspective view of the title compound with the atom numbering scheme. Displacement ellipsoids are at the 50% probability level. Arene bonded hydrogen atoms omitted for clarity.

**Fig. 2.**
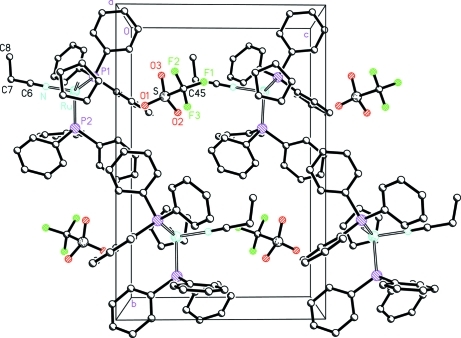
Packing diagram of the title compound viewed down the a axis. H atoms omitted for clarity.

## supplementary crystallographic information

### Comment

Half-sandwich complexes based on the cationic fragment [CpRu]^+^ (Cp = cyclopentadienyl) and complemented by a combination of stabilizing phosphine and labile nitrile ligands are of interest as catalysts in organic chemistry for a variety of useful transformations of unsaturated systems, like C—C bond forming reactions, isomerizations, or C—H activations (Trost *et al.*, 2001). In the context of our research in this area (Rüba *et al.*, 1999, 2002; Mauthner *et al.*, 1999) the title compound was synthesized from [CpRu(PPh_3_)_2_Cl] (Ph = phenyl) by halide abstraction with AgCF_3_SO_3_ in the presence of propionitrile. As an unconventional but efficient method, technical nitromethane was used as the reaction solvent because it provides good solubility of the reactants, already contains the propionitrile needed for the formation of the title compound, and yields a well crystallized product. Fig. 1 shows the asymmetric unit of the title compound. The cationic ruthenium complex exhibits the well known three-legged piano stool geometry with Cp as the seat and Ru—P1 = 2.3585 (4) Å, Ru—P2 = 2.3312 (4) Å, and Ru—N = 2.0422 (15) Å as the legs. The distance between Ru and the centroid of the π-bonded cyclopentadienyl moiety is 1.8579 (8) Å (ring slippage 0.052 Å) and the mean value of the Ru—C bond distances is 2.216 (19) Å. These dimensions agree well with those of the several related complexes, *e.g.* [CpRu(PPh_3_)_2_(NCCH_3_)]BF_4_ (Carreón *et al.*, 1997) or [CpRu(PPh_3_)_2_(NCPh)]PF_6_ (Cordiner *et al.*, 2003). The conformation of the complex in the title compound is irregular and lacks a frequently encountered feature of CpRu(PPh_3_)_2_*X* complexes of having an intramolecular π-π-stacking contact between two phenyl rings opposite to the Cp ring, present for instance in [CpRu(PPh_3_)_2_(NCPh)]PF_6_ (Carreon *et al.*, 1997). A packing diagram of the crystal structure is shown in Fig. 2. Coherence between the complexes and the CF_3_SO_3_ anions is provided by C—H···π, C—H···O and C—H···F interactions, the most significant of which are listed in Table 1. Intermolecular π-π-stacking interactions are absent in the structure.

### Experimental

The synthesis of the title compound was carried out by stirring stoichiometric amounts of [CpRu(PPh_3_)_2_Cl] (Bruce *et al.*, 1982) and AgCF_3_SO_3_ in technical nitromethane at room temperature for several hours. After removing AgCl by filtration through celite, the solution was stored over diethyl ether whereupon well facetted blocky yellow crystals precipitated, showing a distinctly hemimorphic habit with a roof like termination on one side of the *b*-axis (monoclinic, space group *P*2_1_) and a flat base on the other.

### Refinement

All H atoms were placed in calculated positions, with C_arene_—H = 0.95 Å, C_alkyl_—H = 0.99 Å and C_methyl_—H = 0.98 Å and thereafter treated as riding.  *U*_iso_(H) = 1.2*U*_eq_(C_arene_,C_alkyl_) and *U*_iso_(H) = 1.5*U*_eq_(C_methyl_) were used.

### Crystal data

**Table:**

[Ru(C_5_H_5_)(C_3_H_5_N)(C_18_H_15_P)_2_]CF_3_SO_3_	*F*(000) = 916
*M**_r_* = 894.85	*D*_x_ = 1.507 Mg m^−^^3^
Monoclinic, *P*2_1_	Mo *K*α radiation, λ = 0.71073 Å
Hall symbol: P 2yb	Cell parameters from 9696 reflections
*a* = 9.9991 (4) Å	θ = 2.3–30.0°
*b* = 17.2172 (7) Å	µ = 0.59 mm^−^^1^
*c* = 11.4605 (5) Å	*T* = 123 K
β = 92.126 (1)°	Block, yellow
*V* = 1971.64 (14) Å^3^	0.31 × 0.21 × 0.20 mm
*Z* = 2	

### Data collection

**Table:**

Bruker SMART APEX CCD diffractometer	11391 independent reflections
Radiation source: fine-focus sealed tube	11056 reflections with *I* > 2σ(*I*)
graphite	*R*_int_ = 0.023
ω and φ scans	θ_max_ = 30.0°, θ_min_ = 2.1°
Absorption correction: multi-scan (*SADABS*; Bruker, 2003)	*h* = −14→14
*T*_min_ = 0.79, *T*_max_ = 0.89	*k* = −24→24
29537 measured reflections	*l* = −16→16

### Refinement

**Table:**

Refinement on *F*^2^	Secondary atom site location: difference Fourier map
Least-squares matrix: full	Hydrogen site location: inferred from neighbouring sites
*R*[*F*^2^ > 2σ(*F*^2^)] = 0.025	H-atom parameters constrained
*wR*(*F*^2^) = 0.061	*w* = 1/[σ^2^(*F*_o_^2^) + (0.0394*P*)^2^] where *P* = (*F*_o_^2^ + 2*F*_c_^2^)/3
*S* = 1.02	(Δ/σ)_max_ = 0.008
11391 reflections	Δρ_max_ = 0.83 e Å^−^^3^
505 parameters	Δρ_min_ = −0.24 e Å^−^^3^
1 restraint	Absolute structure: Flack (1983), 5488 Friedel pairs
Primary atom site location: structure-invariant direct methods	Flack parameter: −0.038 (12)

### Special details

**Table:**

Geometry. All e.s.d.'s (except the e.s.d. in the dihedral angle between two l.s. planes) are estimated using the full covariance matrix. The cell e.s.d.'s are taken into account individually in the estimation of e.s.d.'s in distances, angles and torsion angles; correlations between e.s.d.'s in cell parameters are only used when they are defined by crystal symmetry. An approximate (isotropic) treatment of cell e.s.d.'s is used for estimating e.s.d.'s involving l.s. planes.
Refinement. Refinement of *F*^2^ against ALL reflections. The weighted *R*-factor *wR* and goodness of fit *S* are based on *F*^2^, conventional *R*-factors *R* are based on *F*, with *F* set to zero for negative *F*^2^. The threshold expression of *F*^2^ > σ(*F*^2^) is used only for calculating *R*-factors(gt) *etc*. and is not relevant to the choice of reflections for refinement. *R*-factors based on *F*^2^ are statistically about twice as large as those based on *F*, and *R*- factors based on ALL data will be even larger.

### Fractional atomic coordinates and isotropic or equivalent isotropic displacement parameters (Å^2^)

**Table:**

	*x*	*y*	*z*	*U*_iso_*/*U*_eq_	
Ru	0.341326 (11)	0.249998 (7)	0.726654 (9)	0.01426 (3)	
C1	0.53032 (17)	0.19188 (12)	0.67577 (17)	0.0228 (4)	
H1	0.5389	0.1642	0.6047	0.027*	
C2	0.49161 (18)	0.15963 (12)	0.78263 (19)	0.0294 (4)	
H2	0.4710	0.1066	0.7960	0.035*	
C3	0.4887 (2)	0.22017 (16)	0.86681 (18)	0.0380 (6)	
H3	0.4643	0.2151	0.9458	0.046*	
C4	0.52913 (19)	0.28964 (14)	0.8112 (2)	0.0350 (5)	
H4	0.5379	0.3392	0.8470	0.042*	
C5	0.55426 (17)	0.27207 (11)	0.69210 (18)	0.0246 (4)	
H5	0.5819	0.3077	0.6345	0.029*	
N	0.25756 (14)	0.23010 (8)	0.56417 (13)	0.0188 (3)	
C6	0.21203 (17)	0.21621 (11)	0.47390 (15)	0.0219 (3)	
C7	0.1453 (2)	0.19964 (15)	0.35985 (17)	0.0342 (5)	
H7A	0.2063	0.2138	0.2972	0.041*	
H7B	0.0644	0.2325	0.3507	0.041*	
C8	0.1054 (2)	0.11580 (17)	0.3454 (2)	0.0469 (6)	
H8A	0.0617	0.1083	0.2683	0.070*	
H8B	0.0432	0.1016	0.4060	0.070*	
H8C	0.1852	0.0829	0.3526	0.070*	
P1	0.15565 (4)	0.18985 (3)	0.80997 (4)	0.01583 (8)	
C9	0.01753 (16)	0.15322 (10)	0.71392 (15)	0.0190 (3)	
C10	−0.03505 (17)	0.20098 (11)	0.62471 (15)	0.0210 (3)	
H10	0.0020	0.2511	0.6134	0.025*	
C11	−0.14125 (18)	0.17569 (13)	0.55243 (18)	0.0265 (4)	
H11	−0.1762	0.2086	0.4922	0.032*	
C12	−0.1962 (2)	0.10270 (14)	0.56792 (19)	0.0283 (4)	
H12	−0.2662	0.0847	0.5165	0.034*	
C13	−0.14818 (19)	0.05593 (13)	0.65905 (19)	0.0292 (4)	
H13	−0.1878	0.0067	0.6720	0.035*	
C14	−0.04185 (19)	0.08125 (11)	0.73145 (18)	0.0241 (4)	
H14	−0.0095	0.0490	0.7935	0.029*	
C15	0.06383 (16)	0.24013 (10)	0.92283 (14)	0.0179 (3)	
C16	0.13777 (18)	0.27803 (11)	1.01128 (15)	0.0219 (3)	
H16	0.2327	0.2778	1.0102	0.026*	
C17	0.0747 (2)	0.31616 (12)	1.10118 (17)	0.0285 (4)	
H17	0.1263	0.3418	1.1609	0.034*	
C18	−0.0637 (2)	0.31647 (13)	1.10291 (18)	0.0311 (4)	
H18	−0.1073	0.3421	1.1643	0.037*	
C19	−0.1390 (2)	0.27944 (13)	1.01531 (18)	0.0294 (4)	
H19	−0.2339	0.2800	1.0168	0.035*	
C20	−0.07604 (16)	0.24154 (12)	0.92526 (15)	0.0221 (3)	
H20	−0.1281	0.2165	0.8653	0.027*	
C21	0.21005 (17)	0.10030 (11)	0.88407 (16)	0.0202 (3)	
C22	0.2503 (2)	0.03787 (12)	0.81551 (19)	0.0263 (4)	
H22	0.2428	0.0416	0.7328	0.032*	
C23	0.3009 (2)	−0.02938 (12)	0.8667 (2)	0.0321 (4)	
H23	0.3278	−0.0714	0.8193	0.039*	
C24	0.3122 (2)	−0.03525 (13)	0.9867 (2)	0.0388 (5)	
H24	0.3475	−0.0813	1.0217	0.047*	
C25	0.2728 (3)	0.02520 (14)	1.0559 (2)	0.0397 (5)	
H25	0.2809	0.0207	1.1384	0.048*	
C26	0.2204 (2)	0.09356 (12)	1.00511 (18)	0.0283 (4)	
H26	0.1921	0.1350	1.0532	0.034*	
P2	0.26534 (4)	0.37799 (2)	0.71997 (4)	0.01567 (8)	
C27	0.25610 (17)	0.43943 (10)	0.84958 (16)	0.0183 (3)	
C28	0.32721 (18)	0.42172 (11)	0.95327 (16)	0.0216 (3)	
H28	0.3744	0.3739	0.9603	0.026*	
C29	0.3294 (2)	0.47364 (13)	1.04651 (17)	0.0279 (4)	
H29	0.3778	0.4610	1.1169	0.034*	
C30	0.2619 (2)	0.54327 (13)	1.03735 (19)	0.0309 (4)	
H30	0.2640	0.5786	1.1011	0.037*	
C31	0.1907 (2)	0.56177 (12)	0.93457 (19)	0.0306 (4)	
H31	0.1438	0.6096	0.9281	0.037*	
C32	0.18831 (19)	0.51047 (11)	0.84190 (17)	0.0242 (4)	
H32	0.1398	0.5236	0.7718	0.029*	
C33	0.09830 (17)	0.39283 (10)	0.65273 (16)	0.0189 (3)	
C34	0.07442 (19)	0.39834 (12)	0.53252 (17)	0.0259 (4)	
H34	0.1479	0.3999	0.4823	0.031*	
C35	−0.0560 (2)	0.40153 (13)	0.48482 (19)	0.0323 (4)	
H35	−0.0710	0.4050	0.4026	0.039*	
C36	−0.1634 (2)	0.39960 (14)	0.5577 (2)	0.0342 (5)	
H36	−0.2522	0.4015	0.5254	0.041*	
C37	−0.14138 (19)	0.39498 (12)	0.6774 (2)	0.0303 (4)	
H37	−0.2153	0.3943	0.7272	0.036*	
C38	−0.01191 (18)	0.39134 (11)	0.72506 (17)	0.0227 (4)	
H38	0.0022	0.3878	0.8073	0.027*	
C39	0.37796 (17)	0.43733 (10)	0.63223 (15)	0.0183 (3)	
C40	0.44301 (18)	0.50304 (11)	0.67898 (17)	0.0216 (3)	
H40	0.4272	0.5188	0.7567	0.026*	
C41	0.53071 (19)	0.54539 (11)	0.61214 (19)	0.0255 (4)	
H41	0.5756	0.5892	0.6452	0.031*	
C42	0.55316 (18)	0.52433 (11)	0.49815 (18)	0.0248 (4)	
H42	0.6118	0.5541	0.4526	0.030*	
C43	0.48912 (18)	0.45894 (11)	0.45029 (17)	0.0226 (3)	
H43	0.5034	0.4445	0.3717	0.027*	
C44	0.40455 (17)	0.41509 (10)	0.51753 (16)	0.0197 (3)	
H44	0.3642	0.3695	0.4856	0.024*	
S	0.46664 (5)	0.27200 (3)	0.20757 (4)	0.02305 (9)	
O1	0.53116 (16)	0.30544 (10)	0.10892 (13)	0.0332 (3)	
O2	0.38633 (16)	0.32535 (10)	0.27204 (14)	0.0360 (3)	
O3	0.40770 (16)	0.19647 (9)	0.18699 (14)	0.0347 (3)	
C45	0.60750 (17)	0.25111 (16)	0.30810 (15)	0.0274 (3)	
F1	0.56484 (15)	0.22620 (9)	0.41086 (11)	0.0455 (4)	
F2	0.68724 (15)	0.19662 (10)	0.26773 (13)	0.0459 (4)	
F3	0.68194 (14)	0.31414 (8)	0.33035 (12)	0.0395 (3)	

### Atomic displacement parameters (Å^2^)

**Table:**

	*U*^11^	*U*^22^	*U*^33^	*U*^12^	*U*^13^	*U*^23^
Ru	0.01224 (5)	0.01522 (5)	0.01522 (5)	0.00110 (5)	−0.00081 (3)	−0.00111 (5)
C1	0.0122 (7)	0.0272 (10)	0.0290 (9)	0.0040 (7)	0.0013 (6)	−0.0069 (8)
C2	0.0172 (8)	0.0293 (10)	0.0418 (11)	0.0081 (7)	0.0006 (7)	0.0111 (8)
C3	0.0190 (8)	0.0745 (17)	0.0199 (9)	0.0123 (9)	−0.0045 (7)	0.0028 (9)
C4	0.0137 (8)	0.0408 (12)	0.0495 (13)	0.0055 (8)	−0.0103 (8)	−0.0256 (10)
C5	0.0125 (7)	0.0238 (9)	0.0374 (10)	−0.0012 (6)	0.0008 (6)	0.0034 (7)
N	0.0171 (6)	0.0182 (7)	0.0211 (7)	−0.0005 (5)	0.0006 (5)	0.0000 (5)
C6	0.0204 (8)	0.0243 (9)	0.0209 (8)	−0.0042 (7)	−0.0002 (6)	−0.0002 (7)
C7	0.0299 (10)	0.0531 (14)	0.0191 (9)	−0.0133 (9)	−0.0054 (7)	0.0005 (9)
C8	0.0344 (12)	0.0593 (17)	0.0468 (14)	−0.0084 (11)	−0.0002 (10)	−0.0244 (13)
P1	0.01420 (18)	0.01554 (19)	0.01770 (19)	0.00049 (15)	0.00011 (13)	−0.00047 (15)
C9	0.0147 (7)	0.0207 (8)	0.0215 (8)	−0.0007 (6)	0.0004 (6)	−0.0041 (6)
C10	0.0151 (7)	0.0230 (8)	0.0247 (8)	0.0001 (6)	−0.0020 (6)	−0.0025 (7)
C11	0.0157 (8)	0.0347 (10)	0.0289 (9)	0.0020 (7)	−0.0035 (6)	−0.0034 (8)
C12	0.0168 (8)	0.0367 (11)	0.0313 (10)	−0.0010 (8)	−0.0022 (7)	−0.0129 (9)
C13	0.0221 (9)	0.0278 (10)	0.0377 (11)	−0.0060 (7)	0.0036 (8)	−0.0104 (8)
C14	0.0222 (9)	0.0220 (9)	0.0281 (9)	−0.0023 (7)	0.0026 (7)	−0.0019 (7)
C15	0.0177 (7)	0.0156 (9)	0.0206 (7)	0.0029 (6)	0.0033 (5)	0.0012 (6)
C16	0.0212 (8)	0.0219 (8)	0.0226 (8)	0.0021 (6)	0.0013 (6)	−0.0006 (7)
C17	0.0338 (10)	0.0281 (10)	0.0235 (9)	0.0030 (8)	0.0026 (8)	−0.0056 (7)
C18	0.0341 (10)	0.0311 (10)	0.0287 (9)	0.0095 (8)	0.0103 (8)	−0.0040 (8)
C19	0.0219 (9)	0.0316 (9)	0.0351 (10)	0.0058 (7)	0.0086 (7)	0.0026 (8)
C20	0.0192 (7)	0.0221 (9)	0.0253 (7)	0.0022 (7)	0.0035 (5)	0.0008 (7)
C21	0.0175 (7)	0.0165 (8)	0.0265 (8)	0.0000 (6)	−0.0005 (6)	0.0016 (6)
C22	0.0248 (9)	0.0207 (9)	0.0336 (10)	0.0026 (7)	0.0065 (8)	−0.0013 (8)
C23	0.0273 (9)	0.0189 (9)	0.0502 (12)	0.0043 (7)	0.0016 (9)	−0.0026 (9)
C24	0.0394 (12)	0.0218 (10)	0.0541 (14)	0.0055 (9)	−0.0135 (10)	0.0076 (10)
C25	0.0531 (15)	0.0310 (11)	0.0340 (12)	0.0062 (11)	−0.0118 (11)	0.0068 (9)
C26	0.0351 (10)	0.0214 (9)	0.0280 (10)	0.0017 (8)	−0.0051 (8)	−0.0001 (7)
P2	0.01508 (18)	0.01485 (18)	0.01694 (18)	0.00050 (15)	−0.00126 (14)	−0.00113 (15)
C27	0.0177 (7)	0.0174 (8)	0.0199 (8)	−0.0006 (6)	0.0009 (6)	−0.0024 (6)
C28	0.0212 (8)	0.0219 (8)	0.0217 (8)	−0.0005 (6)	0.0011 (6)	−0.0021 (6)
C29	0.0293 (10)	0.0345 (11)	0.0198 (8)	−0.0008 (8)	−0.0007 (7)	−0.0052 (8)
C30	0.0295 (10)	0.0344 (11)	0.0289 (10)	−0.0022 (8)	0.0034 (8)	−0.0146 (8)
C31	0.0304 (10)	0.0237 (9)	0.0377 (11)	0.0052 (8)	0.0025 (8)	−0.0106 (8)
C32	0.0242 (9)	0.0213 (9)	0.0271 (9)	0.0032 (7)	−0.0017 (7)	−0.0028 (7)
C33	0.0165 (7)	0.0151 (8)	0.0248 (8)	0.0006 (6)	−0.0035 (6)	−0.0003 (6)
C34	0.0232 (8)	0.0270 (9)	0.0269 (9)	−0.0022 (7)	−0.0059 (7)	0.0048 (7)
C35	0.0282 (9)	0.0326 (11)	0.0351 (11)	0.0002 (8)	−0.0133 (8)	0.0047 (9)
C36	0.0182 (9)	0.0282 (11)	0.0550 (14)	0.0020 (8)	−0.0121 (9)	−0.0015 (10)
C37	0.0184 (8)	0.0254 (10)	0.0468 (12)	0.0011 (7)	−0.0004 (8)	−0.0048 (9)
C38	0.0202 (8)	0.0187 (9)	0.0290 (9)	−0.0001 (7)	−0.0011 (7)	−0.0017 (7)
C39	0.0166 (7)	0.0162 (7)	0.0221 (8)	0.0010 (6)	−0.0004 (6)	0.0013 (6)
C40	0.0205 (8)	0.0186 (8)	0.0258 (8)	−0.0013 (6)	0.0001 (6)	−0.0030 (6)
C41	0.0202 (8)	0.0193 (9)	0.0368 (10)	−0.0037 (7)	0.0012 (7)	−0.0012 (7)
C42	0.0207 (8)	0.0206 (9)	0.0331 (10)	0.0018 (7)	0.0028 (7)	0.0066 (7)
C43	0.0221 (8)	0.0219 (9)	0.0242 (9)	0.0033 (7)	0.0048 (7)	0.0014 (7)
C44	0.0190 (8)	0.0168 (8)	0.0233 (8)	−0.0001 (6)	−0.0005 (6)	−0.0011 (6)
S	0.0246 (2)	0.0236 (2)	0.02063 (19)	−0.00254 (16)	−0.00403 (16)	−0.00122 (15)
O1	0.0360 (8)	0.0380 (8)	0.0252 (7)	−0.0037 (7)	−0.0027 (6)	0.0067 (6)
O2	0.0315 (7)	0.0410 (9)	0.0352 (8)	0.0058 (7)	−0.0030 (6)	−0.0090 (7)
O3	0.0391 (8)	0.0279 (7)	0.0365 (8)	−0.0110 (7)	−0.0083 (6)	−0.0012 (6)
C45	0.0287 (8)	0.0288 (8)	0.0243 (7)	−0.0020 (10)	−0.0050 (6)	0.0039 (10)
F1	0.0506 (8)	0.0623 (10)	0.0230 (6)	−0.0067 (7)	−0.0065 (5)	0.0146 (6)
F2	0.0417 (7)	0.0473 (9)	0.0477 (9)	0.0181 (7)	−0.0103 (6)	−0.0071 (7)
F3	0.0374 (7)	0.0382 (7)	0.0417 (7)	−0.0098 (6)	−0.0163 (6)	−0.0013 (6)

### Geometric parameters (Å, °)

**Table:**

Ru—N	2.0422 (15)	C22—C23	1.385 (3)
Ru—C4	2.1901 (19)	C22—H22	0.9500
Ru—C3	2.2001 (19)	C23—C24	1.379 (4)
Ru—C5	2.2129 (17)	C23—H23	0.9500
Ru—C1	2.2348 (17)	C24—C25	1.375 (4)
Ru—C2	2.2403 (18)	C24—H24	0.9500
Ru—P2	2.3312 (4)	C25—C26	1.406 (3)
Ru—P1	2.3585 (4)	C25—H25	0.9500
C1—C2	1.412 (3)	C26—H26	0.9500
C1—C5	1.412 (3)	P2—C27	1.8288 (18)
C1—H1	0.9500	P2—C33	1.8312 (17)
C2—C3	1.421 (3)	P2—C39	1.8458 (18)
C2—H2	0.9500	C27—C28	1.396 (3)
C3—C4	1.421 (4)	C27—C32	1.399 (3)
C3—H3	0.9500	C28—C29	1.393 (3)
C4—C5	1.430 (3)	C28—H28	0.9500
C4—H4	0.9500	C29—C30	1.378 (3)
C5—H5	0.9500	C29—H29	0.9500
N—C6	1.140 (2)	C30—C31	1.390 (3)
C6—C7	1.473 (3)	C30—H30	0.9500
C7—C8	1.505 (4)	C31—C32	1.381 (3)
C7—H7A	0.9900	C31—H31	0.9500
C7—H7B	0.9900	C32—H32	0.9500
C8—H8A	0.9800	C33—C34	1.393 (3)
C8—H8B	0.9800	C33—C38	1.404 (3)
C8—H8C	0.9800	C34—C35	1.396 (3)
P1—C15	1.8312 (17)	C34—H34	0.9500
P1—C21	1.8331 (18)	C35—C36	1.385 (3)
P1—C9	1.8451 (17)	C35—H35	0.9500
C9—C14	1.392 (3)	C36—C37	1.384 (3)
C9—C10	1.399 (2)	C36—H36	0.9500
C10—C11	1.392 (2)	C37—C38	1.388 (3)
C10—H10	0.9500	C37—H37	0.9500
C11—C12	1.386 (3)	C38—H38	0.9500
C11—H11	0.9500	C39—C40	1.401 (2)
C12—C13	1.390 (3)	C39—C44	1.404 (2)
C12—H12	0.9500	C40—C41	1.392 (3)
C13—C14	1.394 (3)	C40—H40	0.9500
C13—H13	0.9500	C41—C42	1.382 (3)
C14—H14	0.9500	C41—H41	0.9500
C15—C16	1.394 (2)	C42—C43	1.397 (3)
C15—C20	1.400 (2)	C42—H42	0.9500
C16—C17	1.392 (3)	C43—C44	1.388 (3)
C16—H16	0.9500	C43—H43	0.9500
C17—C18	1.385 (3)	C44—H44	0.9500
C17—H17	0.9500	S—O2	1.4418 (16)
C18—C19	1.387 (3)	S—O1	1.4422 (15)
C18—H18	0.9500	S—O3	1.4435 (16)
C19—C20	1.391 (3)	S—C45	1.8221 (17)
C19—H19	0.9500	C45—F2	1.326 (3)
C20—H20	0.9500	C45—F3	1.335 (3)
C21—C26	1.392 (3)	C45—F1	1.338 (2)
C21—C22	1.399 (3)		
			
N—Ru—C4	140.51 (8)	C18—C17—C16	119.51 (19)
N—Ru—C3	150.17 (8)	C18—C17—H17	120.2
C4—Ru—C3	37.77 (10)	C16—C17—H17	120.2
N—Ru—C5	103.32 (7)	C17—C18—C19	120.24 (18)
C4—Ru—C5	37.89 (8)	C17—C18—H18	119.9
C3—Ru—C5	63.12 (8)	C19—C18—H18	119.9
N—Ru—C1	90.52 (6)	C18—C19—C20	120.26 (18)
C4—Ru—C1	62.32 (7)	C18—C19—H19	119.9
C3—Ru—C1	62.35 (8)	C20—C19—H19	119.9
C5—Ru—C1	37.03 (8)	C19—C20—C15	120.18 (17)
N—Ru—C2	113.12 (7)	C19—C20—H20	119.9
C4—Ru—C2	62.27 (9)	C15—C20—H20	119.9
C3—Ru—C2	37.32 (9)	C26—C21—C22	118.91 (18)
C5—Ru—C2	62.03 (7)	C26—C21—P1	122.79 (15)
C1—Ru—C2	36.78 (7)	C22—C21—P1	118.20 (15)
N—Ru—P2	90.37 (4)	C23—C22—C21	120.8 (2)
C4—Ru—P2	89.60 (6)	C23—C22—H22	119.6
C3—Ru—P2	116.96 (7)	C21—C22—H22	119.6
C5—Ru—P2	98.42 (5)	C24—C23—C22	119.9 (2)
C1—Ru—P2	133.82 (5)	C24—C23—H23	120.0
C2—Ru—P2	151.67 (6)	C22—C23—H23	120.0
N—Ru—P1	89.61 (4)	C25—C24—C23	120.3 (2)
C4—Ru—P1	129.24 (7)	C25—C24—H24	119.9
C3—Ru—P1	96.78 (6)	C23—C24—H24	119.9
C5—Ru—P1	157.66 (5)	C24—C25—C26	120.4 (2)
C1—Ru—P1	126.52 (6)	C24—C25—H25	119.8
C2—Ru—P1	96.25 (5)	C26—C25—H25	119.8
P2—Ru—P1	99.661 (15)	C21—C26—C25	119.7 (2)
C2—C1—C5	108.67 (17)	C21—C26—H26	120.2
C2—C1—Ru	71.82 (10)	C25—C26—H26	120.2
C5—C1—Ru	70.64 (10)	C27—P2—C33	100.86 (8)
C2—C1—H1	125.7	C27—P2—C39	99.93 (8)
C5—C1—H1	125.7	C33—P2—C39	105.01 (8)
Ru—C1—H1	123.5	C27—P2—Ru	123.12 (6)
C1—C2—C3	108.27 (19)	C33—P2—Ru	115.90 (6)
C1—C2—Ru	71.40 (10)	C39—P2—Ru	109.66 (6)
C3—C2—Ru	69.80 (11)	C28—C27—C32	118.34 (17)
C1—C2—H2	125.9	C28—C27—P2	121.71 (14)
C3—C2—H2	125.9	C32—C27—P2	119.57 (14)
Ru—C2—H2	124.5	C29—C28—C27	120.41 (18)
C4—C3—C2	107.44 (18)	C29—C28—H28	119.8
C4—C3—Ru	70.73 (11)	C27—C28—H28	119.8
C2—C3—Ru	72.87 (11)	C30—C29—C28	120.40 (19)
C4—C3—H3	126.3	C30—C29—H29	119.8
C2—C3—H3	126.3	C28—C29—H29	119.8
Ru—C3—H3	121.8	C29—C30—C31	119.85 (18)
C3—C4—C5	108.24 (19)	C29—C30—H30	120.1
C3—C4—Ru	71.50 (11)	C31—C30—H30	120.1
C5—C4—Ru	71.92 (10)	C32—C31—C30	119.93 (19)
C3—C4—H4	125.9	C32—C31—H31	120.0
C5—C4—H4	125.9	C30—C31—H31	120.0
Ru—C4—H4	122.4	C31—C32—C27	121.06 (18)
C1—C5—C4	107.36 (18)	C31—C32—H32	119.5
C1—C5—Ru	72.33 (10)	C27—C32—H32	119.5
C4—C5—Ru	70.19 (10)	C34—C33—C38	118.42 (16)
C1—C5—H5	126.3	C34—C33—P2	123.09 (14)
C4—C5—H5	126.3	C38—C33—P2	118.21 (13)
Ru—C5—H5	122.8	C33—C34—C35	120.89 (19)
C6—N—Ru	177.51 (15)	C33—C34—H34	119.6
N—C6—C7	176.6 (2)	C35—C34—H34	119.6
C6—C7—C8	113.1 (2)	C36—C35—C34	119.8 (2)
C6—C7—H7A	109.0	C36—C35—H35	120.1
C8—C7—H7A	109.0	C34—C35—H35	120.1
C6—C7—H7B	109.0	C37—C36—C35	120.06 (19)
C8—C7—H7B	109.0	C37—C36—H36	120.0
H7A—C7—H7B	107.8	C35—C36—H36	120.0
C7—C8—H8A	109.5	C36—C37—C38	120.3 (2)
C7—C8—H8B	109.5	C36—C37—H37	119.8
H8A—C8—H8B	109.5	C38—C37—H37	119.8
C7—C8—H8C	109.5	C37—C38—C33	120.53 (19)
H8A—C8—H8C	109.5	C37—C38—H38	119.7
H8B—C8—H8C	109.5	C33—C38—H38	119.7
C15—P1—C21	102.61 (8)	C40—C39—C44	118.47 (16)
C15—P1—C9	101.49 (8)	C40—C39—P2	121.50 (14)
C21—P1—C9	101.04 (8)	C44—C39—P2	119.98 (13)
C15—P1—Ru	120.08 (6)	C41—C40—C39	120.36 (17)
C21—P1—Ru	109.41 (6)	C41—C40—H40	119.8
C9—P1—Ru	119.45 (6)	C39—C40—H40	119.8
C14—C9—C10	118.55 (16)	C42—C41—C40	120.70 (18)
C14—C9—P1	122.15 (14)	C42—C41—H41	119.7
C10—C9—P1	119.19 (13)	C40—C41—H41	119.7
C11—C10—C9	120.57 (18)	C41—C42—C43	119.62 (18)
C11—C10—H10	119.7	C41—C42—H42	120.2
C9—C10—H10	119.7	C43—C42—H42	120.2
C12—C11—C10	120.31 (19)	C44—C43—C42	120.00 (18)
C12—C11—H11	119.8	C44—C43—H43	120.0
C10—C11—H11	119.8	C42—C43—H43	120.0
C11—C12—C13	119.61 (18)	C43—C44—C39	120.79 (17)
C11—C12—H12	120.2	C43—C44—H44	119.6
C13—C12—H12	120.2	C39—C44—H44	119.6
C12—C13—C14	120.0 (2)	O2—S—O1	114.91 (10)
C12—C13—H13	120.0	O2—S—O3	115.21 (10)
C14—C13—H13	120.0	O1—S—O3	115.05 (10)
C9—C14—C13	120.83 (19)	O2—S—C45	103.52 (10)
C9—C14—H14	119.6	O1—S—C45	102.52 (9)
C13—C14—H14	119.6	O3—S—C45	103.11 (11)
C16—C15—C20	118.72 (16)	F2—C45—F3	107.67 (16)
C16—C15—P1	117.93 (12)	F2—C45—F1	107.2 (2)
C20—C15—P1	123.34 (13)	F3—C45—F1	106.62 (16)
C17—C16—C15	121.08 (17)	F2—C45—S	112.32 (13)
C17—C16—H16	119.5	F3—C45—S	111.88 (16)
C15—C16—H16	119.5	F1—C45—S	110.84 (12)
			
N—Ru—C1—C2	−130.06 (13)	C10—C11—C12—C13	2.5 (3)
C4—Ru—C1—C2	79.87 (15)	C11—C12—C13—C14	−2.5 (3)
C3—Ru—C1—C2	37.00 (14)	C10—C9—C14—C13	2.4 (3)
C5—Ru—C1—C2	118.20 (17)	P1—C9—C14—C13	178.46 (14)
P2—Ru—C1—C2	138.93 (11)	C12—C13—C14—C9	0.0 (3)
P1—Ru—C1—C2	−40.16 (14)	C21—P1—C15—C16	77.57 (15)
N—Ru—C1—C5	111.75 (12)	C9—P1—C15—C16	−178.21 (14)
C4—Ru—C1—C5	−38.32 (13)	Ru—P1—C15—C16	−43.98 (16)
C3—Ru—C1—C5	−81.19 (14)	C21—P1—C15—C20	−101.66 (16)
C2—Ru—C1—C5	−118.20 (17)	C9—P1—C15—C20	2.57 (17)
P2—Ru—C1—C5	20.73 (14)	Ru—P1—C15—C20	136.79 (14)
P1—Ru—C1—C5	−158.36 (10)	C20—C15—C16—C17	0.5 (3)
C5—C1—C2—C3	1.0 (2)	P1—C15—C16—C17	−178.74 (15)
Ru—C1—C2—C3	−60.35 (13)	C15—C16—C17—C18	0.0 (3)
C5—C1—C2—Ru	61.37 (13)	C16—C17—C18—C19	−0.4 (3)
N—Ru—C2—C1	56.32 (13)	C17—C18—C19—C20	0.2 (3)
C4—Ru—C2—C1	−80.02 (14)	C18—C19—C20—C15	0.4 (3)
C3—Ru—C2—C1	−118.44 (18)	C16—C15—C20—C19	−0.7 (3)
C5—Ru—C2—C1	−36.94 (12)	P1—C15—C20—C19	178.51 (14)
P2—Ru—C2—C1	−87.45 (16)	C15—P1—C21—C26	−21.73 (18)
P1—Ru—C2—C1	148.57 (11)	C9—P1—C21—C26	−126.30 (17)
N—Ru—C2—C3	174.76 (12)	Ru—P1—C21—C26	106.84 (16)
C4—Ru—C2—C3	38.41 (13)	C15—P1—C21—C22	162.15 (15)
C5—Ru—C2—C3	81.50 (14)	C9—P1—C21—C22	57.57 (16)
C1—Ru—C2—C3	118.44 (18)	Ru—P1—C21—C22	−69.29 (15)
P2—Ru—C2—C3	30.99 (19)	C26—C21—C22—C23	−0.7 (3)
P1—Ru—C2—C3	−92.99 (13)	P1—C21—C22—C23	175.61 (15)
C1—C2—C3—C4	−1.3 (2)	C21—C22—C23—C24	−0.1 (3)
Ru—C2—C3—C4	−62.70 (13)	C22—C23—C24—C25	0.4 (4)
C1—C2—C3—Ru	61.36 (13)	C23—C24—C25—C26	0.0 (4)
N—Ru—C3—C4	106.38 (19)	C22—C21—C26—C25	1.1 (3)
C5—Ru—C3—C4	37.78 (11)	P1—C21—C26—C25	−175.01 (17)
C1—Ru—C3—C4	79.63 (12)	C24—C25—C26—C21	−0.8 (4)
C2—Ru—C3—C4	116.10 (17)	N—Ru—P2—C27	163.43 (8)
P2—Ru—C3—C4	−47.99 (12)	C4—Ru—P2—C27	−56.06 (10)
P1—Ru—C3—C4	−152.47 (11)	C3—Ru—P2—C27	−28.99 (10)
N—Ru—C3—C2	−9.7 (2)	C5—Ru—P2—C27	−93.04 (9)
C4—Ru—C3—C2	−116.10 (17)	C1—Ru—P2—C27	−105.49 (10)
C5—Ru—C3—C2	−78.32 (13)	C2—Ru—P2—C27	−49.49 (14)
C1—Ru—C3—C2	−36.47 (12)	P1—Ru—P2—C27	73.77 (7)
P2—Ru—C3—C2	−164.09 (10)	N—Ru—P2—C33	38.91 (8)
P1—Ru—C3—C2	91.43 (12)	C4—Ru—P2—C33	179.42 (9)
C2—C3—C4—C5	1.2 (2)	C3—Ru—P2—C33	−153.51 (9)
Ru—C3—C4—C5	−62.94 (13)	C5—Ru—P2—C33	142.44 (8)
C2—C3—C4—Ru	64.10 (13)	C1—Ru—P2—C33	129.99 (9)
N—Ru—C4—C3	−131.38 (14)	C2—Ru—P2—C33	−174.01 (13)
C5—Ru—C4—C3	−117.16 (18)	P1—Ru—P2—C33	−50.75 (7)
C1—Ru—C4—C3	−79.71 (13)	N—Ru—P2—C39	−79.71 (7)
C2—Ru—C4—C3	−37.96 (12)	C4—Ru—P2—C39	60.79 (9)
P2—Ru—C4—C3	138.52 (12)	C3—Ru—P2—C39	87.86 (9)
P1—Ru—C4—C3	36.34 (14)	C5—Ru—P2—C39	23.81 (8)
N—Ru—C4—C5	−14.22 (19)	C1—Ru—P2—C39	11.36 (9)
C3—Ru—C4—C5	117.16 (18)	C2—Ru—P2—C39	67.36 (13)
C1—Ru—C4—C5	37.45 (12)	P1—Ru—P2—C39	−169.38 (6)
C2—Ru—C4—C5	79.20 (13)	C33—P2—C27—C28	149.20 (15)
P2—Ru—C4—C5	−104.32 (12)	C39—P2—C27—C28	−103.26 (16)
P1—Ru—C4—C5	153.50 (10)	Ru—P2—C27—C28	18.20 (18)
C2—C1—C5—C4	−0.3 (2)	C33—P2—C27—C32	−37.97 (17)
Ru—C1—C5—C4	61.82 (12)	C39—P2—C27—C32	69.56 (16)
C2—C1—C5—Ru	−62.11 (12)	Ru—P2—C27—C32	−168.97 (12)
C3—C4—C5—C1	−0.5 (2)	C32—C27—C28—C29	0.3 (3)
Ru—C4—C5—C1	−63.21 (13)	P2—C27—C28—C29	173.25 (15)
C3—C4—C5—Ru	62.67 (13)	C27—C28—C29—C30	−0.3 (3)
N—Ru—C5—C1	−72.64 (12)	C28—C29—C30—C31	0.2 (3)
C4—Ru—C5—C1	116.60 (18)	C29—C30—C31—C32	−0.2 (3)
C3—Ru—C5—C1	78.94 (13)	C30—C31—C32—C27	0.3 (3)
C2—Ru—C5—C1	36.69 (12)	C28—C27—C32—C31	−0.4 (3)
P2—Ru—C5—C1	−165.04 (10)	P2—C27—C32—C31	−173.44 (16)
P1—Ru—C5—C1	51.2 (2)	C27—P2—C33—C34	142.10 (16)
N—Ru—C5—C4	170.76 (13)	C39—P2—C33—C34	38.62 (17)
C3—Ru—C5—C4	−37.66 (14)	Ru—P2—C33—C34	−82.54 (16)
C1—Ru—C5—C4	−116.60 (18)	C27—P2—C33—C38	−43.99 (16)
C2—Ru—C5—C4	−79.90 (14)	C39—P2—C33—C38	−147.47 (14)
P2—Ru—C5—C4	78.37 (13)	Ru—P2—C33—C38	91.37 (14)
P1—Ru—C5—C4	−65.4 (2)	C38—C33—C34—C35	−0.6 (3)
N—Ru—P1—C15	−127.55 (7)	P2—C33—C34—C35	173.26 (16)
C4—Ru—P1—C15	60.22 (10)	C33—C34—C35—C36	0.3 (3)
C3—Ru—P1—C15	81.66 (9)	C34—C35—C36—C37	0.4 (3)
C5—Ru—P1—C15	106.34 (15)	C35—C36—C37—C38	−0.7 (3)
C1—Ru—P1—C15	142.09 (9)	C36—C37—C38—C33	0.4 (3)
C2—Ru—P1—C15	119.23 (9)	C34—C33—C38—C37	0.3 (3)
P2—Ru—P1—C15	−37.24 (6)	P2—C33—C38—C37	−173.94 (15)
N—Ru—P1—C21	114.31 (8)	C27—P2—C39—C40	8.39 (16)
C4—Ru—P1—C21	−57.92 (10)	C33—P2—C39—C40	112.57 (15)
C3—Ru—P1—C21	−36.48 (9)	Ru—P2—C39—C40	−122.27 (13)
C5—Ru—P1—C21	−11.80 (16)	C27—P2—C39—C44	−174.34 (14)
C1—Ru—P1—C21	23.95 (9)	C33—P2—C39—C44	−70.16 (15)
C2—Ru—P1—C21	1.09 (9)	Ru—P2—C39—C44	55.00 (15)
P2—Ru—P1—C21	−155.39 (6)	C44—C39—C40—C41	0.7 (3)
N—Ru—P1—C9	−1.30 (8)	P2—C39—C40—C41	177.97 (14)
C4—Ru—P1—C9	−173.53 (10)	C39—C40—C41—C42	1.2 (3)
C3—Ru—P1—C9	−152.09 (9)	C40—C41—C42—C43	−1.3 (3)
C5—Ru—P1—C9	−127.40 (15)	C41—C42—C43—C44	−0.6 (3)
C1—Ru—P1—C9	−91.66 (9)	C42—C43—C44—C39	2.6 (3)
C2—Ru—P1—C9	−114.52 (9)	C40—C39—C44—C43	−2.6 (3)
P2—Ru—P1—C9	89.01 (7)	P2—C39—C44—C43	−179.92 (14)
C15—P1—C9—C14	−88.98 (16)	O2—S—C45—F2	174.08 (17)
C21—P1—C9—C14	16.49 (17)	O1—S—C45—F2	−66.11 (19)
Ru—P1—C9—C14	136.43 (13)	O3—S—C45—F2	53.69 (18)
C15—P1—C9—C10	87.08 (15)	O2—S—C45—F3	−64.69 (15)
C21—P1—C9—C10	−167.45 (14)	O1—S—C45—F3	55.12 (16)
Ru—P1—C9—C10	−47.51 (16)	O3—S—C45—F3	174.93 (14)
C14—C9—C10—C11	−2.4 (3)	O2—S—C45—F1	54.2 (2)
P1—C9—C10—C11	−178.57 (14)	O1—S—C45—F1	173.99 (17)
C9—C10—C11—C12	−0.1 (3)	O3—S—C45—F1	−66.21 (19)

### Hydrogen-bond geometry (Å, °)

**Table:**

*D*—H···*A*	*D*—H	H···*A*	*D*···*A*	*D*—H···*A*
C1—H1···F1	0.95	2.49	3.125 (2)	125
C3—H3···O1^i^	0.95	2.50	3.154 (3)	126
C7—H7A···O3	0.99	2.43	3.346 (3)	153
C28—H28···O1^i^	0.95	2.56	3.328 (2)	138
C44—H44···O2	0.95	2.58	3.209 (2)	124

Symmetry codes: (i) *x*, *y*, *z*+1.
